# Adaptation mechanisms of low-phosphorus stress in plants: physiological responses, molecular regulation, and future perspectives

**DOI:** 10.3389/fpls.2026.1877859

**Published:** 2026-07-30

**Authors:** Junhao Zhang, Ao Pan, Zhangqiang Song, Xiaohui Liu, Jun Zhang, Guoping Zhang

**Affiliations:** 1 Institute of Crop Science, Zhejiang University, Hangzhou, China; 2 Key Laboratory of Cotton Breeding and Cultivation in Huang-Huai-Hai Plain, Ministry of Agriculture and Rual Affairs, Institute of Industrial Crops, Shandong Academy of Agricultural Science, Jinan, China

**Keywords:** low-Pi, phosphate transporters, root exudates, root structure, signaling pathway, SPX-PHR, transcription factors

## Abstract

Phosphorus (P) is essential for plant growth and development. Although soils contain abundant total P, about 70% of global arable land is deficient in available inorganic phosphate (Pi), severely restricting sustainable agricultural production. To date, numerous physiological and molecular mechanisms underlying plant adaptation to low-Pi stress have been elucidated. In this review, we provide an overview of recent advances in plant adaptation to low-Pi stress at both the physiological and molecular levels, including root plasticity and hormonal regulation, root exudate-mediated Pi acquisition, metabolic adaptation such as sugar metabolism, membrane lipid remodeling, and secondary metabolite accumulation, as well as arbuscular mycorrhizal (AM) symbiosis. Furthermore, we summarize the molecular regulatory networks governing plant responses to low-Pi stress, covering phosphate transporters, SPX-PHR signaling, transcription factors, non-coding RNAs, and epigenetic modifications. The interaction between low-Pi signaling and other signaling pathways is also discussed. This review synthesizes recent advances in adaptive mechanisms across multiple regulatory levels and discusses strategies for breeding P-efficient crops to support sustainable agriculture.

## 1 Introduction

Plant growth and development largely depend on phosphorus (P), a crucial macronutrient constituting around 0.2-1.1% of plant dry biomass (Péret et al., 2014). P in plants is majorly present in inorganic and organic forms, with organic P making up approximately 85% of total P and inorganic P occupying nearly 15% (Yang et al., 2017). Moreover, P is fundamental to plant growth and development through forming nucleic acids for genetic information, comprising phospholipids that mediate membrane transport, and mediate ATP production, promoting photosynthesis, respiration, and carbohydrate metabolism (Raghothama and Karthikeyan, 2005).

Lower P availability, caused by lower mobility and stronger P fixation, contributes to ~ 70% of croplands impacted by P deficiency, though most soils contain higher total P concentration (**Supplementary Figure 1**) (López-Arredondo et al., 2014; Herrera-Estrella and López-Arredondo, 2016; McDowell et al., 2024). When growing under low inorganic phosphate (Pi) conditions, photosynthesis, energy metabolism, and signal transduction are strongly prohibited, causing plant growth restriction (Carstensen et al., 2018). To alleviate the P deficiency in arable land, large amounts of phosphate fertilizers are applied, but their low utilization efficiency exacerbates phosphate rock depletion and runoff, resulting in eutrophication and other environmental problems (Elser and Bennett, 2011).

Plants possess sophisticated mechanisms for tackling low-Pi stress involving physiological, biochemical, and molecular responses through long-period evolution. Explaining these mechanisms is essential because it provides both theoretical support and valuable genetic resources for enhancing crop P-use efficiency. This paper comprehensively reviews plant low-Pi stress responses and adaptation via physiological and molecular regulation. Furthermore, we also dissect future research directions and strategies for low-Pi adaptation improvement in plants.

## 2 Physiological adaptations to low-Pi stress and the underlying molecular basis

**Figure 1:**
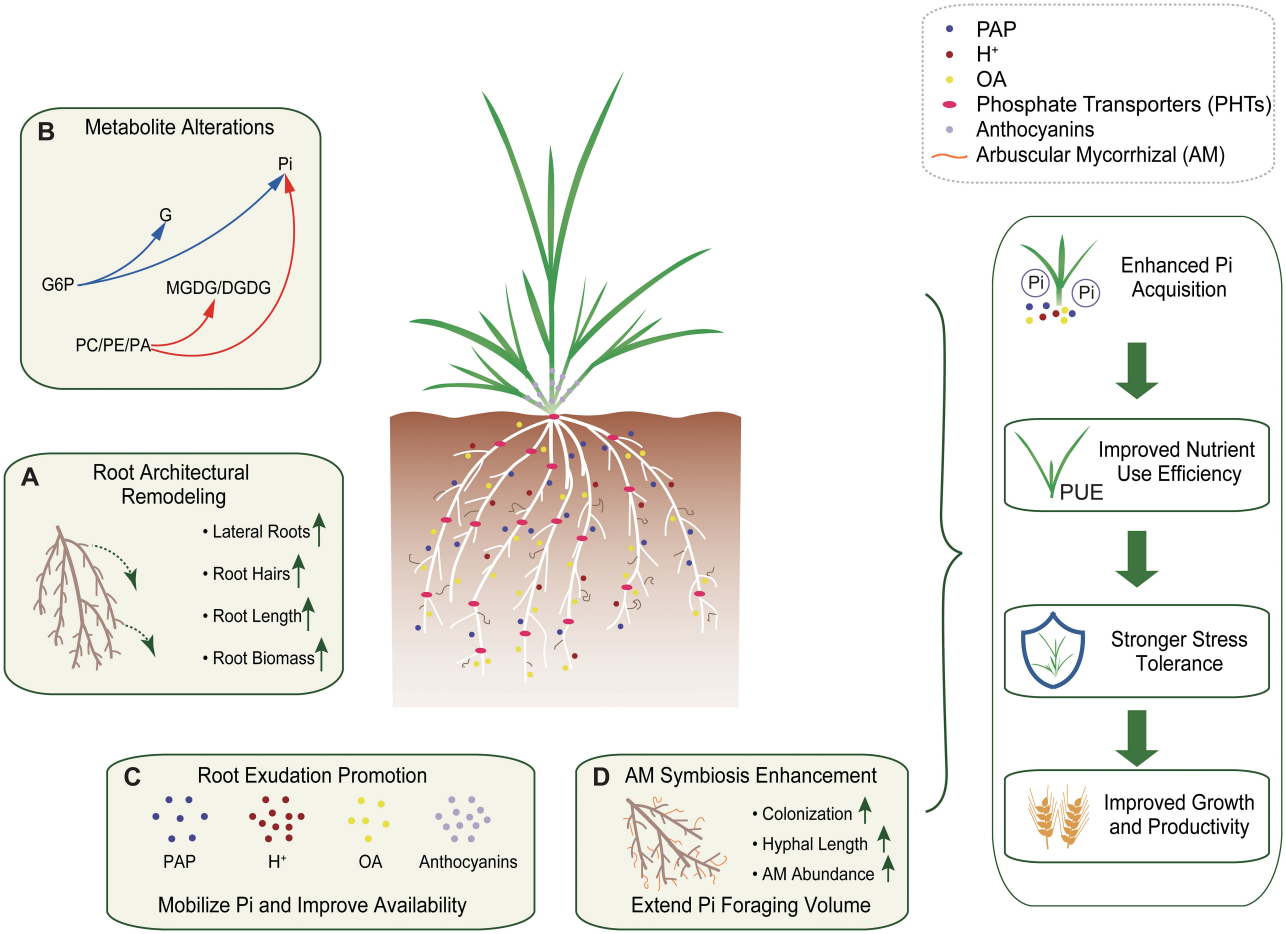
Schematic overview of plant adaptation to low-Pi stress. Low Pi induces coordinated changes in root architecture **(A)**, metabolism **(B)**, root exudation **(C)**, and AM symbiosis **(D)**, resulting in enhanced Pi acquisition, improved nutrient use efficiency, stronger stress tolerance, and increased plant growth and productivity.

When plants subjected to low-Pi environments, they adopt various physiological and metabolic adaptive strategies encompassing root system remodeling, increased root exudation, metabolic reprogramming, and arbuscular mycorrhizal (AM) symbiosis (**Figure 1**) (Wang et al., 2019). By coordinating these strategies, plants can improve Pi uptake and utilization under Pi starvation, thereby strengthening their Pi-deficient adaptation.

### 2.1 Remodeling of the root system architecture and its hormonal regulation

The plant root system is the main organ for inorganic P uptake from the soil. Numerous studies have discovered that plants root system is altered to deal with Pi starvation through enhanced density and length of lateral roots and root hairs (**Figure 1**). Pi starvation promotes root hair development, causing elongation in *Arabidopsis thaliana* and increased formation in rice (*Oryza sativa*) and maize (*Zea mays*) (Niu et al., 2013; Sun et al., 2014; Guo et al., 2025). These changes increase the root absorptive area and the root/shoot biomass ratio to improve phosphorus uptake and utilization (López-Bucio et al., 2002; López-Arredondo et al., 2014). Balzergue et al. (2017) observed that low Pi prohibits primary root growth via two distinct root apex responses: an early STOP1-associated inhibition of cell elongation, and a slower LPR1-dependent reduction in meristem activity. Pi starvation inhibits primary root growth and remodels root architecture through local signaling pathways in *Arabidopsis*, whereas it facilitates primary root elongation but reduces lateral root density in rice (Puga et al., 2024).

Low-Pi-induced root system remodeling in plants is finely regulated by hormone signaling, with auxin functioning as a central regulator. Auxin-related gene families, including AUX/LAX, ARF, PIN, and TAA/YUCCA, have been reported to modulate root architecture by different regulatory mechanisms under low Pi environment. AUX1 plays a role in auxin redistribution to the root hair differentiation zone, leading to root hair elongation during Pi starvation (Bhosale et al., 2018). *OsARF16* knockout restricts lateral root and root hair development under low-Pi conditions, while *ARF19* promotes auxin-mediated root hair elongation during Pi starvation in *Arabidopsis* (Shen et al., 2013; Bhosale et al., 2018). PIN proteins regulate root architecture through directional auxin transport, but are prohibited under Pi starvation, consequently disrupting auxin flux and impeding primary root development (Adamowski and Friml, 2015; Zhang et al., 2026). TAA/YUCCA-dependent auxin biosynthesis orchestrates root architecture under Pi deficiency through controlling lateral root and root hair formation in rice (Kang et al., 2025).

Moreover, other hormones such as strigolactones, ethylene, and cytokinins also participate in regulating root development under low Pi stress. Low Pi promotes strigolactone biosynthesis, facilitating RSA remodeling and Pi uptake via the D14–SDEL1–SPX4 pathway in rice (Gu et al., 2023). Ethylene antagonizes MYB30 to mediate RSL4 and other PSR genes via EIN3, governing root hair growth and phosphate uptake under Pi deficiency (Xiao et al., 2021). *OsCKX2*, encoding the cytokinin oxidase/dehydrogenase, inhibits rice tolerance to Pi deficiency via modulating cytokinin, thereby altering Pi transporter expression, remodeling membrane lipid and glycolysis to limit P uptake and growth under low-Pi conditions (Yan et al., 2022). Collectively, Pi starvation enhances root hair formation and lateral root development, increasing the absorptive surface for phosphorus uptake. Different species exhibit contrasting responses: *Arabidopsis* displays primary root inhibition, whereas rice and maize generally maintain or enhance primary root growth, controlled by auxin in coordination with strigolactone, ethylene, and cytokinin signaling.

### 2.2 Promotion of root exudation for organic P mobilization

When growing under low-Pi environment, plant roots promote the synthesis and secretion of root exudates, thereby facilitating the mineralization of external organic P and improving available P concentration in the soil (**Figure 1**). Low-Pi stress facilitates cluster root formation and stimulates exudation of protons, organic acids, and acid phosphatases, causing rhizosphere acidification and improved Pi availability and uptake. Intercropping experiments with faba bean (*Vicia faba*) and maize have indicated that faba bean roots mobilize sparingly soluble phosphorus by H^+^ release, thereby enhancing P-use efficiency in maize (Li et al., 2007).

Low-Pi stress significantly facilitates the synthesis and exudation of organic acids, including malate and citrate, in plant roots. These organic acids can convert sparingly soluble phosphorus into available forms by means of chelation, ion exchange, and dissolution. The ALMT (aluminum-activated malate transporter) and MATE family transporters were discovered to modulate malate and citrate secretion, respectively (Tovkach et al., 2013; Peng et al., 2018). STOP1, a transcription factor, regulates organic acid secretion by triggering the efflux transporters ALMT1 and MATE in *Arabidopsis thaliana* (Sawaki et al., 2009). Tian et al. (2021b) discovered that low-Pi conditions promoted STOP1 protein accumulation, thereby improving *ALMT* expression and modulating the mobilization of sparingly soluble phosphorus and the release of Pi.

Plants secrete large amounts of purple acid phosphatases (PAPs) into the root-associated soil under low-Pi environment to catalyze the hydrolysis of organic phosphorus, consequently increasing inorganic phosphate availability. The *PAP* gene family is conserved across plant species, including rice, *Arabidopsis*, and soybean (*Glycine max*), and functions in phosphorus acquisition by solubilizing organic phosphorus through APase-mediated hydrolysis. In rice, overexpression of *OsPAP10a*, *OsPAP10c*, and *OsPAP21b* display higher activity of APase and enhanced exogenous organic phosphorus utilization efficiency (Tian et al., 2012; Mehra et al., 2017; Deng et al., 2020). In *Arabidopsis*, several PAPs play a role in organic P mobilization under low Pi stress. *AtPAP10* is activated by Pi starvation and can hydrolyze a variety of P-containing organic compounds (Wang et al., 2011), while *atpap12* and *atpap26* mutations reduce the organic P utilization efficiency (Robinson et al., 2012). In soybean, PAPs have also been shown to participate in phosphorus acquisition and utilization. Heterologous overexpression of *AtPAP15* in soybean upregulates both APase and phytase activities, leading to improved phosphorus-use efficiency compared to the wild type (Wang et al., 2009b). Some *PAP* members in soybean, such as *GmPAP17*, *GmPAP7a*, and *GmPAP7b*, positively regulate APase activity and improve P-use efficiency under Pi starvation (Zhu et al., 2020; Xu et al., 2022). These studies suggest that *PAP* genes are important for organic phosphorus mobilization and plant adaptation to Pi deficiency.

### 2.3 Adjustment of metabolic pathways

Plant metabolism is reprogrammed in a sophisticated but integrated manner by Pi starvation to maintain Pi homeostasis and sustain normal physiological functions. In carbohydrate metabolism, photosynthesis, which includes chlorophyll biosynthesis, the Calvin cycle, and photorespiration, is strongly suppressed by low-Pi stress (Morcuende et al., 2007). Meanwhile, the ATP-dependent glycolytic pathway is inhibited, while alternative glycolytic routes are enhanced to optimize ATP consumption and promote Pi recycling (**Figure 1**) (Plaxton and Tran, 2011). Glucose-6-phosphate (G6P) and fructose-6-phosphate (F6P), key sugar phosphate metabolites, are dramatically decreased by Pi starvation and function as metabolic markers of low-Pi (**Figure 1**) (Huang et al., 2008). Pi starvation severely prohibits sucrose degradation in maize shoots, whereas sucrose catabolism remains stable in roots, thereby increasing hexose-to-sucrose ratio that facilitates root growth and development (Xiao et al., 2024).

Lipid metabolism is primarily influenced by membrane lipid remodeling when living under Pi deficiency. In order to sustain cellular Pi homeostasis under Pi starvation, plant membrane phospholipids (e.g., PC, PE, PA) are degraded and remodeled into non-phosphorus lipids including MGDG and DGDG (**Figure 1**) (Andersson et al., 2005; Tjellström et al., 2010). Some investigations have found that *OsDGD1* affects the membrane lipid remodeling process by adjusting DGDG content, thereby regulating phosphate use efficiency (Pandey et al., 2025). Besides, a growing body of research has pointed out that *SQD1*, encoding UDP-sulfoquinovose synthase, function as a primary contributor to this process (Yu et al., 2002; Sun et al., 2020, Sun et al., 2021). Moreover, the upregulation of *NPC4* and *GCS* modulate sphingolipid remodeling under Pi deprivation via adjusting sphingolipid hydrolysis and inducing glucosylceramide synthesis, respectively (Yang et al., 2021, Yang et al., 2025).

Secondary metabolites such as anthocyanins and flavonoids, which alleviate Pi stress-induced damage in plants, are also increased by Pi deficiency (**Figure 1**) (Pant et al., 2015). Anthocyanin biosynthesis under Pi starvation is primarily governed by PAP1, with SPX4 participating in this process through its interaction with PAP1 and modulation of Pi signaling (He et al., 2021b). Subsequent investigations have proved that the transcription factor WRKY33 interacts with PHR1 and undergoes degradation under Pi deficiency, thereby relieving its repression on *DFR* and promoting anthocyanin biosynthesis (Tao et al., 2024). Pi starvation contributes to coordinated reprogramming of carbohydrate, lipid, and secondary metabolism, but how these pathways are quantitatively balanced across different species remains unknown.

### 2.4 Arbuscular mycorrhizal symbiosis

Currently, some investigations have proved that AM symbiosis can moderately lessen low-Pi stress, subsequently promoting Pi uptake in crops and strengthening their adaptation to Pi deficiency (**Figure 1**). Maize growth and Pi acquisition are facilitated under low-Pi environment, and Pi deficiency is slightly mitigated after inoculation with AM fungi (AMF) (Liang et al., 2022). Another study has also shown that AMF inoculation under low-Pi conditions dramatically promotes growth and tolerance in wheat genotypes with low P-use efficiency, whereas the impact on Pi-efficient genotypes remains negligible (Sun et al., 2025), suggesting that mycorrhizal symbiosis may impart genotype-dependent benefits on wheat low-Pi tolerance. Strigolactone (SL, GR24) induces AM symbiosis in rice, thereby enhancing Pi absorption and growth under low-Pi stress, and improving soil function in conjunction with AMF (Mitra et al., 2024).

AM fungi are crucial for plant Pi absorption and can contribute substantially to total P uptake in crops (Ferrol et al., 2019). Elucidating how AM symbiosis enhances Pi deficiency adaptation in plants has been considered one of the key research areas. Some studies have identified that the phosphate transporters OsPHT1;11 and OsPHT1;13 influence rice AM development (**Supplementary Table 1**). Among them, OsPHT1;11 is a key component involved in Pi uptake via the AM symbiosis, whereas OsPHT1;13 does not directly participate in AM-mediated Pi acquisition but may function as a sensor of local Pi status to facilitate AM development (**Supplementary Table 1**) (Yang et al., 2012). Similarly, AMF improves *PHT* gene expression in alfalfa, including *MtPT4*, thereby enhancing plant adaptation under Pi starvation conditions (Nguyen et al., 2019). Besides, PHOSPHATE STARVATION RESPONSE 2 (PHR2) promotes AM symbiosis in rice through regulating pre-contact signaling events, root colonization process, and AM-related gene expression, associating AM symbiosis with low-Pi signaling (Das et al., 2022). These findings preliminarily suggest that AM symbiosis interacts with Pi-deficiency signaling to strengthen low-Pi adaptation in plants when growing under low-Pi conditions. AM symbiosis can improve plant Pi uptake and low-P tolerance, but its effectiveness relies heavily on genotype. Future works should focus on elucidating the genetic basis of AM responsiveness and its interaction with Pi signaling pathways.

## 3 Molecular regulatory networks of low-phosphate responses in plants

Beyond the physiological and metabolic adaptations described above, plants possess an elaborate intracellular signaling and gene regulatory network that perceives Pi status and orchestrates downstream responses.

### 3.1 Phosphate transporter systems (PHTs and PHOs)

Phosphate transporters (PHTs) are major contributors to the acquisition and transportation of inorganic phosphate in plants. Plant phosphate transporters are grouped into five families (PHT1–PHT5), each characterized by their different subcellular localization and function (**Supplementary Table 1**).

The PHT1 family, comprising plasma membrane–localized proteins with 12 transmembrane domains, functions as H^+^/Pi symporters and contributes significantly to Pi absorption from soil environments and its translocation to aboveground tissues in plants (Nussaume et al., 2011). Rice and *Arabidopsis* possess 13 (OsPHT1;1–OsPHT1;13, also named OsPT1–OsPT13) and 9 (AtPHT1;1–AtPHT1;9) PHT1 transporters, respectively (**Supplementary Table 1**) (Paszkowski et al., 2002; Nussaume et al., 2011). Several members of the OsPHT1 family, including OsPHT1;1-OsPHT1;6, are essential for regulating Pi absorption and translocation (**Supplementary Table 1**) (Paszkowski et al., 2002; Ai et al., 2009; Sun et al., 2012; Ye et al., 2015; Chang et al., 2019). OsPHT1;7 not only act as a regulator of source-to-sink Pi redistribution but also influences anther Pi accumulation and pollen fertility in rice (**Supplementary Table 1**) (Dai et al., 2022). OsPHT1;8 mediates Pi homeostasis and internal Pi distribution, thereby affecting plant growth and development (**Supplementary Table 1**) (Jia et al., 2011). Furthermore, *OsPHT1;9* and *OsPHT1;10*, induced by Pi deprivation, redundantly function as Pi uptake transporters (**Supplementary Table 1**) (Wang et al., 2014c). Except for *OsPHT1;1* and *OsPHT1;8*, the mRNA levels of these genes are enhanced by low-Pi environment (**Supplementary Table 1**) (Paszkowski et al., 2002; Ai et al., 2009; Jia et al., 2011; Sun et al., 2012; Wang et al., 2014c; Ye et al., 2015; Chang et al., 2019; Dai et al., 2022). Findings from a recent study indicate that the cryo-EM structures of OsPHT1;11 in both ligand-bound and apo states, indicating that conformational changes between outward- and inward-facing states are connected to Pi transport activity, thereby advancing understanding of the molecular basis of plant phosphate regulation (Du et al., 2026). In *Arabidopsis*, only AtPHT1;1, AtPHT1;4, AtPHT1;5, AtPHT1;8, and AtPHT1;9 have been functionally characterized (**Supplementary Table 1**) (Shin et al., 2004; Smith et al., 2011; Remy et al., 2012), among which AtPHT1;1 and AtPHT1;4 function as the predominant contributors to Pi absorption in roots (**Supplementary Table 1**) (Shin et al., 2004). AtPHT1;5 operates as a source-to-sink Pi transporter in plants, sustaining Pi homeostasis (**Supplementary Table 1**) (Smith et al., 2011), while AtPHT1;8 and AtPHT1;9 contribute to root Pi uptake; notably, AtPHT1;9 does not mediate root-to-shoot Pi translocation (**Supplementary Table 1**) (Remy et al., 2012).

PHT2, a chloroplast envelope-localized protein, mainly regulates Pi distribution in the plant (**Supplementary Table 1**) (Versaw and Harrison, 2002). Both OsPHT2;1 and AtPHT2;1 regulate Pi import into chloroplasts to maintain chloroplast Pi homeostasis, while only OsPHT2;1 has been confirmed in the maintenance of normal photosynthesis (**Supplementary Table 1**) (Versaw and Harrison, 2002; Liu et al., 2020). The mitochondria-localized protein PHT3 mediates Pi exchange across the mitochondrial membrane (**Supplementary Table 1**) (Poirier and Bucher, 2002). PHT4 regulates phosphate trafficking among the cytosol, chloroplasts, heterotrophic plastids, and the Golgi apparatus, with its expression remaining nearly unchanged under Pi starvation (**Supplementary Table 1**) (Guo et al., 2008). The PHT5/VPT family, involved in vacuolar Pi transport, affects vacuolar Pi import and storage (**Supplementary Table 1**) (Liu et al., 2016). Moreover, both BnA09PHT5;1b and BnCnPHT5;1b, homologs of AtPHT5;1 in *Arabidopsis*, contribute to intracellular Pi homeostasis and promote rapeseed growth and development (Han et al., 2022).

PHO1, belonging to the SPX-EXS family, plays a key role in systemic Pi transport. *pho1* mutants maintain normal roots Pi concentration in *Arabidopsis*, but exhibit a marked reduction in shoot Pi levels and a severe Pi-deficiency symptom (Hamburger et al., 2002). PHO1 is not consistently retained at the plasma membrane but instead undergoes constitutive clathrin-mediated endocytic cycling, which plays a critical role in root-to-shoot Pi export and whole-plant Pi homeostasis (Vetal and Poirier, 2023). PHTs and PHO1 constitute a conserved transport system coordinating Pi uptake, intracellular redistribution, and long-distance movement across plant species. However, current functional evidence is still mainly based on rice and *Arabidopsis*, and regulatory divergence among crops remains to be fully explored.

### 3.2 The SPX-PHR core signaling module

**Figure 2:**
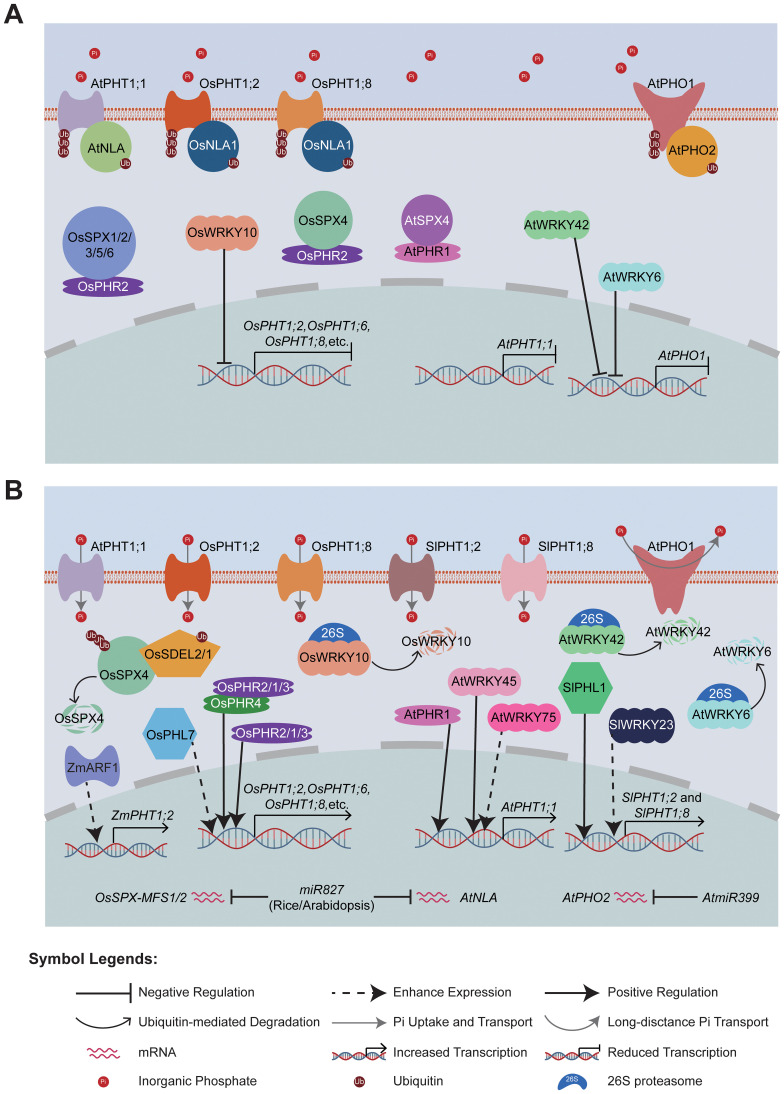
Molecular mechanisms underlying plant responses to Pi-sufficient **(A)** and Pi-deficient **(B)** environments. **(A)** Under Pi-sufficient conditions, SPX proteins inhibit PHR activity, thereby suppressing PSI gene expression and Pi acquisition. Meanwhile, NLA/PHO2-mediated ubiquitination and WRKY-dependent repression restrict Pi transporter abundance or activity to sustain Pi balance. **(B)** Under low-Pi conditions, release of negative regulation, including SPX-mediated PHR inhibition release, activates the PHR-mediated signaling pathway, accompanied by enhanced PSI gene expression and PHT1 transporter activity for Pi uptake, transport, and redistribution under Pi starvation.

The SPX-PHR signaling network is a core module of phosphate signaling in plants. Under sufficient Pi availability, SPX proteins suppress PHR transcription factors, while Pi starvation releases PHRs from SPX-mediated repression (**Figure 2**) (Wang et al., 2026). A conserved protein domain known as SPX, named after Syg1, Pho81, and Xpr1, is implicated in phosphate signaling pathways as well as Pi homeostasis in plants (Secco et al., 2012). Under sufficient external Pi supply, OsSPX1/2 tightly bind OsPHR2 to negatively regulate the phosphate starvation response, whereas this interaction weakens with declining Pi levels (**Figure 2**; **Supplementary Table 2**) (Wang et al., 2009a; Puga et al., 2014; Wang et al., 2014d). Although *OsSPX4* expression is not enhanced by Pi deficiency, its protein is degraded under Pi-limited environment, thus de-repressing OsPHR2 and subsequently activating phosphate starvation responses (**Figure 2B**; **Supplementary Table 2**) (Lv et al., 2014). Similarly, HvSPX4 restricts HvPHR2 activity to sustain Pi homeostasis, and fragment deletions in the *HvSPX4* promoter reduce its expression, thereby enhancing barley adaptation to low-Pi conditions (Zhang et al., 2025). In rice, OsSPX4 is ubiquitinated by SDELs proteins under Pi-deficient conditions, promoting its degradation and thereby releasing PHR2 (**Figure 2B**) (Ruan et al., 2019). OsSPX6 impedes PHR2 interaction with the P1BS motif in a tissue-specific manner, being degraded in leaves but accumulating in roots (**Supplementary Table 2**) (Zhong et al., 2018). Similarly to other SPX members, OsSPX3 and OsSPX5 redundantly suppress low-Pi responses through direct and indirect inhibition of PHR2 activity (**Figure 2A**; **Supplementary Table 2**), while *OsSPX3* negatively regulates *OsSPX5* expression in shoots under Pi deficiency (**Supplementary Table 2**) (Wang et al., 2009c; Shi et al., 2014). When it comes to the function of AtSPXs, Duan et al. (2008) discovered AtSPX1 and AtSPX3 positive regulate Pi starvation response, with AtSPX3 potentially exhibiting negative feedback regulation on AtSPX1 (**Supplementary Table 2**). AtSPX4 negatively regulates shoot low-Pi responses via sequestering PHR1 in the cytosol and modulating both PHR1-dependent and PHR1-independent transcriptional networks (**Supplementary Table 2**) (Osorio et al., 2019).

As members of the MYB-CC transcription factor family, PHRs are exemplified by PHR1 (OsPHR2 in rice), which serves as a core regulator of phosphate homeostasis and triggers phosphate starvation-induced (PSI) genes, such as PHT1 family members, by interacting with the P1BS cis-element (GNATATNC) located in target gene promoters (**Figure 2B**; **Supplementary Table 3**) (Bari et al., 2006; Hu et al., 2025). In *Arabidopsis*, *phr1* mutants are sensitive to Pi deficiency, characterized by reduced root crown size, downregulated PSI gene expression, and decreased anthocyanin accumulation, whereas *PHR1* overexpression increases the expression of PSI gene, promotes Pi accumulation, and confers improved low-Pi conditions adaptation (**Supplementary Table 3**) (Nilsson et al., 2007). PHR1 has been characterized as an essential integrator of Pi signaling with other nutrient signals, including sulfur, zinc, and iron (**Supplementary Table 3**) (Bournier et al., 2013; Khan et al., 2014). OsPHR1, OsPHR2, OsPHR3, and OsPHR4 cooperatively modulate phosphate signaling pathways and homeostatic regulation in rice (**Figure 2B**; **Supplementary Table 3**) (Guo et al., 2015; Ruan et al., 2017). Of these, OsPHR2, together with OsPHR1 and OsPHR3, functions redundantly to stimulate PSI gene expression, thereby promoting low-Pi responses (**Figure 2B**; **Supplementary Table 3**) (Guo et al., 2015; Sun et al., 2018). However, these three transcription factors exhibit distinct DNA-binding affinities for downstream promoters, variable expression across tissues and developmental stages, and varying interactions with target genes (Guo et al., 2015). In contrast to OsPHR1–3, OsPHR4 interacts with OsPHR1–3 and seems to be activated by them, playing a role in low-Pi responses and Pi homeostasis (**Figure 2B**; **Supplementary Table 3**) (Ruan et al., 2017).

Classical SPX proteins, other SPX domain-containing families, such as SPX-MFS, SPX-RING, and SPX-EXS, are also involved in phosphate signaling and homeostasis, each possessing a shared SPX domain at the N-terminus but distinct C-terminal domains (MFS, RING, or EXS) (**Supplementary Table 2**) (Lin et al., 2010; Secco et al., 2012). Under Pi deficiency, *OsSPX-MFS1* is downregulated whereas *OsSPX-MFS2* is upregulated, and *OsSPX-MFS1* is predominantly expressed in shoots, where they potentially function as phosphate transporters that regulate leaf Pi homeostasis (**Supplementary Table 2**) (Lin et al., 2010; Wang et al., 2012). In contrast, OsSPX-MFS3 is a major regulator that governs vacuolar Pi efflux and sustains Pi homeostasis (**Supplementary Table 2**) (Wang et al., 2015; Guo et al., 2023).

The SPX-RING family, acting as a member of E3 ubiquitin ligases (e.g., NLA1/NLA2), drives degradation of phosphate transporters via ubiquitination. AtNLA orchestrates the endocytosis and phosphate transporters degradation such as PHT1;1 through ubiquitination in *Arabidopsis*, thereby ensuring Pi homeostasis (**Supplementary Table 2**) (Lin et al., 2013; Park et al., 2014). AtNLA regulates nitrate-dependent Pi homeostasis (**Supplementary Table 2**) (Kant et al., 2011). Similarly, phosphate transporters OsPT2 and OsPT8 can be ubiquitinated by OsNLA1 in rice (**Figure 2A**; **Supplementary Table 2**) (Yue et al., 2017). Predominantly expressed in root pericycle and xylem parenchyma cells, AtPHO1 is responsible for Pi loading into the xylem for root-to-shoot transport (**Supplementary Table 2**) (Hamburger et al., 2002). PHO2, a ubiquitin-conjugating enzyme, targets AtPHO1 for ubiquitin-mediated suppression; Pi deficiency destabilizes PHO2 transcripts, thereby increasing AtPHO1 abundance and Pi transport, while pho2 mutants exhibit shoot Pi overaccumulation caused by impaired shoot-to-root remobilization (**Figure 2B**; **Supplementary Table 2**) (Bari et al., 2006; Chiou et al., 2006; Liu et al., 2012). In addition to AtPHO1, a total of ten paralogs (AtPHO1;H1–AtPHO1;H10) have been documented, but only a few of them have been functionally characterized. AtPHO1;H1 controls Pi efflux via a channel-like mechanism, which mediates xylem loading and root-to-shoot phosphate translocation (**Supplementary Table 2**) (Fang et al., 2025). AtPHO1;H4 functions in cryptochrome signaling, seed development, photoperiod control, and flowering, while AtPHO1;H10 is implicated in the response to diverse biotic and abiotic stresses (**Supplementary Table 2**) (Wang et al., 2004; Kang and Ni, 2006; Ribot et al., 2008; Zhou and Ni, 2009). OsPHO1;2 coordinates Pi transport with grain filling while enhancing starch synthesis-related enzyme activity in rice, thereby improving phosphorus use efficiency (**Supplementary Table 2**) (Ma et al., 2021). OsPHO1;2 modulates leaf-to-grain Pi allocation to maintain leaf Pi supply, sustaining Pi homeostasis and relieving Pi deficiency-induced inhibition of photosynthesis (**Supplementary Table 2**) (Ma et al., 2024). OsPHO1;3, stimulated by Pi deficiency, facilitates phloem-mediated Pi transport to actively growing tissues such as young and upper leaves, thus contributing to maintaining plant Pi homeostasis (**Supplementary Table 2**) (Yan et al., 2024). OsPHO1;1 coordinates phosphate, zinc, and iron homeostasis in rice through regulating Fe transport responding to Pi–Zn deficiency, impacting iron distribution between roots and shoots (**Supplementary Table 2**) (Saenchai et al., 2016).

The SPX–PHR module is conserved in plants, but its regulation differs among species in aspects such as protein stability, tissue specificity, and feedback control. However, most evidence of SPX–PHR regulation remains at the level of description, with limited cross-species quantitative work connecting these variations to P-use efficiency. This highlights the requirement for crop-level functional studies and comparative analyses to identify SPX–PHR regulatory variants useful for enhancing phosphorus-use efficiency in breeding.

### 3.3 Transcription factor regulatory networks

Besides PHRs, other MYB family members also influence the low-Pi response in plants. Some members of the PHL (PHR1-LIKE) subfamily, such as PHL1, also promote downstream PSI gene expression under low-Pi conditions (**Figure 2B**; **Supplementary Table 3**). They function either redundantly or synergistically with PHR1 in regulating low-Pi stress responses, with PHL1 serving as a major co-regulator of PHR1 (**Supplementary Table 3**) (Sun et al., 2016; Wang et al., 2023d). PHL4 also participates in Pi starvation responses and to function redundantly with PHR1, but with a relative weaker contribution than PHL1 and without synergistic interaction with it, instead predominantly acting to prohibit growth and development in plants (**Supplementary Table 3**) (Wang et al., 2018). SlPHL1 directly interacts with the P1BS cis-element, inducing downstream Pi starvation-responsive genes such as *SlPht1;2* and *SlPht1;8* in tomato, thereby eliciting Pi deficiency response (**Figure 2B**; **Supplementary Table 3**) (Zhang et al., 2021). Pi starvation also enhances *OsPHL7* expression, which facilitates Pi uptake and root development by transcriptionally activating PSI genes such as OsPHT1;2 and OsPHT1;6, thus improving rice adaptation to both low-Pi conditions and salt stress (**Figure 2B**; **Supplementary Table 3**) (Yang et al., 2024). Overexpression of *MYB62*, an R2R3-type MYB transcription factor, suppresses PSI gene expression and alters root structure, Pi uptake, and acid phosphatase activity (**Supplementary Table 3**) (Devaiah et al., 2009). MYB1 knockout lines result in an improvement in Pi absorption and accumulation in rice (**Supplementary Table 3**) (Gu et al., 2017). OsMYB110, an R2R3-type MYB transcription factor induced by OsPHR2, negatively affects plant height in rice, as knockout of *OsMYB110* results in higher plant height and grain yield regardless of external Pi supply (**Supplementary Table 3**) (Wang et al., 2024).

The WRKY protein family is also crucial for low-Pi stress responses. WRKY75, the first identified WRKY transcription factor associated with Pi nutrition, is upregulated by Pi starvation and serves as a positive regulator of PSI genes such as those encoding PHT1 transporters (**Figure 2B**; **Supplementary Table 3**) (Devaiah et al., 2007a). WRKY6, together with its homolog WRKY42, represses root-to-shoot Pi translocation through W-box-mediated suppression of *PHO1* promoter activity under Pi-sufficient conditions (**Figure 2A**; **Supplementary Table 3**) (Chen et al., 2009; Su et al., 2015). *WRKY45* is upregulated by Pi deficiency and contribute to Pi-deficiency tolerance through binding to the two W-box of *PHT1;1* promoter and enhancing *PHT1;1* expression (**Figure 2B**; **Supplementary Table 3**) (Wang et al., 2014a). WRKY33 negatively regulates low-Pi responses by repressing ALMT1-mediated root growth and anthocyanin accumulation (**Supplementary Table 3**) (Shen et al., 2021; Tao et al., 2024), and the underlying regulatory mechanism is discussed in Section 2.3. OsWRKY10 directly inhibits *OsPHT1;2* expression under Pi-sufficient environment to restrict Pi uptake in rice, whereas under low Pi environment, it is targeted for degradation through the 26S proteasome pathway, thus removing its repression on *OsPHT1;2* and potentially promoting Pi uptake (**Figure 2**; **Supplementary Table 3**) (Wang et al., 2023b). SlWRKY23 promotes the Pi-starvation response in tomato by sustaining primary root and lateral root growth, enhancing downstream PSR-related gene expression, and activating acid phosphatase genes (**Figure 2B**; **Supplementary Table 3**) (Zhao et al., 2026).

Several bHLH family members also regulate Pi deficiency responses. *OsPTF1* overexpression induces Pi uptake-related genes together with improved root system architecture (longer roots and greater root surface area), thereby enhancing tolerance to low-Pi stress tolerance in rice (**Supplementary Table 3**) (Yi et al., 2005). On the contrary, under low-Pi stress, degradation of bHLH32, suppressing Pi starvation, results in the upregulation of PSI genes (**Supplementary Table 3**) (Chen et al., 2007, Chen et al., 2008). *OsbHLH6* overexpression triggers root hair elongation, increases Pi content, and upregulates PSI genes (**Supplementary Table 3**) (He et al., 2021a). PIF3, a member of the bHLH phytochrome-interacting factor subfamily, enhances plant growth under Pi starvation, positively regulating Pi starvation and integrating carbon status signals such as sucrose availability with low-Pi signaling (**Supplementary Table 3**) (Chen et al., 2025). The zinc-finger protein ZAT6 is stimulated by low-Pi stress, yet it functions as a negative regulator, as its overexpression represses phosphate uptake capacity (**Supplementary Table 3**) (Devaiah et al., 2007b). Some AP2/ERF families are also required for regulating plant Pi starvation responses. AtERF70 impacts lateral and primary root development, as well as Pi accumulation (**Supplementary Table 3**) (Ramaiah et al., 2014), while GmERF1 in soybean binds to GmWRKY6 to restrict phosphate transporter gene expression, thereby reducing phosphate uptake (**Supplementary Table 3**) (Wang et al., 2023a). Another AP2/ERF transcription factor, GmAP2, enhances plant Pi starvation adaptation via modulating low Pi stress responsive genes including *AtPHR1* and by promoting lateral root number (**Supplementary Table 3**) (Li et al., 2025). BZR1 and BES1, transcription factors associated with brassinosteroid signaling, negatively regulate the low-Pi response (**Supplementary Table 3**) (Liu et al., 2023). Low Pi conditions inhibit *OsARF12* expression, an auxin response factor, leading to decreased Pi content in rice (**Supplementary Table 3**) (Wang et al., 2014b). ZmARF1 activates *ZmLBD1* expression to accelerate lateral root development and positively regulates core Pi starvation genes expression, including *ZmPHR1* and *ZmPHT1;2*, thus directly improving low-P stress tolerance in maize (**Figure 2B**; **Supplementary Table 3**) (Wu et al., 2024). Pi-responsive transcription factors are conserved across plants but differ in regulatory strength, hierarchy, and developmental outputs among species. These MYB/PHR-, WRKY-, bHLH-, AP2/ERF-, and hormone-related factors form interconnected networks integrating Pi signaling with growth and environmental responses, with notable species divergence. Combining multi-omics approaches with genetic validation in diverse crops may uncover key transcription factors underlying phosphate adaptation and facilitate the development of strategies for enhancing phosphorus-use efficiency and low Pi adaptation.

### 3.4 Non-coding RNA

Non-coding RNAs act as key regulators in plant response to low-Pi stress and have been broadly characterized. miR399, identified as the first miRNA, shows improved expression under Pi starvation, and it regulates Pi acquisition and redistribution through targeting the mRNA of *PHO2*, thus reducing its expression (**Figure 2B**) (Fujii et al., 2005; Bari et al., 2006). miR827 modulates *AtNLA* in *Arabidopsis* or *OsSPX-MFS1* and *OsSPX-MFS2* in rice, consequently regulating phosphate homeostasis (**Figure 2B**) (Pant et al., 2009; Lin et al., 2010). miR156, miR2111, and miR778 are also involved in Pi starvation and play roles in plant Pi deficiency tolerance (Hsieh et al., 2009). Maize miR1432, negatively regulating low-Pi stress, potentially reduced low-Pi stress tolerance by restricting organic acid secretion through mediating *ZmCML21* (Pei et al., 2026). Some long non-coding RNAs, such as *AtIPS1/AT4*, *OsIPS1*, and *OsIPS2*, serve as indicators of low-Pi signaling in plants and are markedly upregulated under Pi deficiency (Hou et al., 2005; Shin et al., 2006). Maize *PILNCR2* is a low-Pi-inducible lncRNA that forms RNA duplexes with *ZmPHT1* transcripts, interfering with miR399-mediated cleavage, thereby maintaining *PHT1* mRNA stability and inducing its expression, consequently enhancing maize low-Pi tolerance and Pi uptake (Wang et al., 2023c). Non-coding RNAs are involved in Pi starvation adaptation through diverse regulatory modes, while their functional divergence among plant species is still only partly understood. Identifying key ncRNA and its target modules in crops will be important for uncovering novel regulatory strategies for improving Pi-use efficiency and adaptation to low-Pi adaptation conditions.

### 3.5 Epigenetic mechanism

Several studies have demonstrated that epigenetic modifications affect low-Pi stress responses in plants and maintain phosphate homeostasis. When exposed to low-Pi conditions, PHR1 modulates DNA methyltransferases-related gene expression, thereby influencing DNA methylation status and altering phosphate starvation response (PSR) gene expression (Yong-Villalobos et al., 2016). OTU5, a key epigenetic regulator, mediates low-Pi adaptation via DNA and histone methylation, where histone methylation controls root hair and root architecture–related genes, and DNA methylation ensures genome stability under low-Pi environment (Yen et al., 2017). Low-Pi stress may modulate PSR gene expression via DNA methylation changes, majorly happening in nearby transposable elements (TE) instead of directly regulating transcription or alternative splicing, indicating that DNA methylation mainly affects TE distribution and has limited straight impact on PSR responses (Tian et al., 2021a). Chromatin-mediated regulation, including histone acetylation, is involved in low-Pi responses, where Pi deficiency improves H3 acetylation to enhance root remodeling genes, while HDC1 (Histone Deacetylase Complex 1) represses their expression and hdc1 mutants display improved low-Pi phenotypes including suppressed root growth and elevated root hair density (Xu et al., 2020). The chromatin remodeler BRAHMA (BRM) recruits the HDA6-HDC1 complex to regulate H3 deacetylation of *LPR1/2* genes, thereby restricting iron-dependent local Pi deficiency responses, whereas under Pi deficiency, BRM degradation enhances H3 acetylation, activates *LPR1/2* transcription, and triggers root system remodeling (Li et al., 2022). Recent findings suggest that under low-Pi conditions, m^6^A modifications stabilize PSR-related mRNAs, including *PHR1*, at the post-transcriptional level, constituting a positive feedback loop that enhances systemic Pi starvation responses and support Pi deficiency adaptation in plants (Liu et al., 2025). Although epigenetic regulation integrates DNA methylation, histone marks, and m6A in Pi responses, their coordination at cell-type resolution is still unclear. Further studies are required to connect epigenetic aspects with PHR1-centered networks under low-Pi stress.

### 3.6 Cross-regulation between Pi signaling and multiple signaling pathways

**Figure 3:**
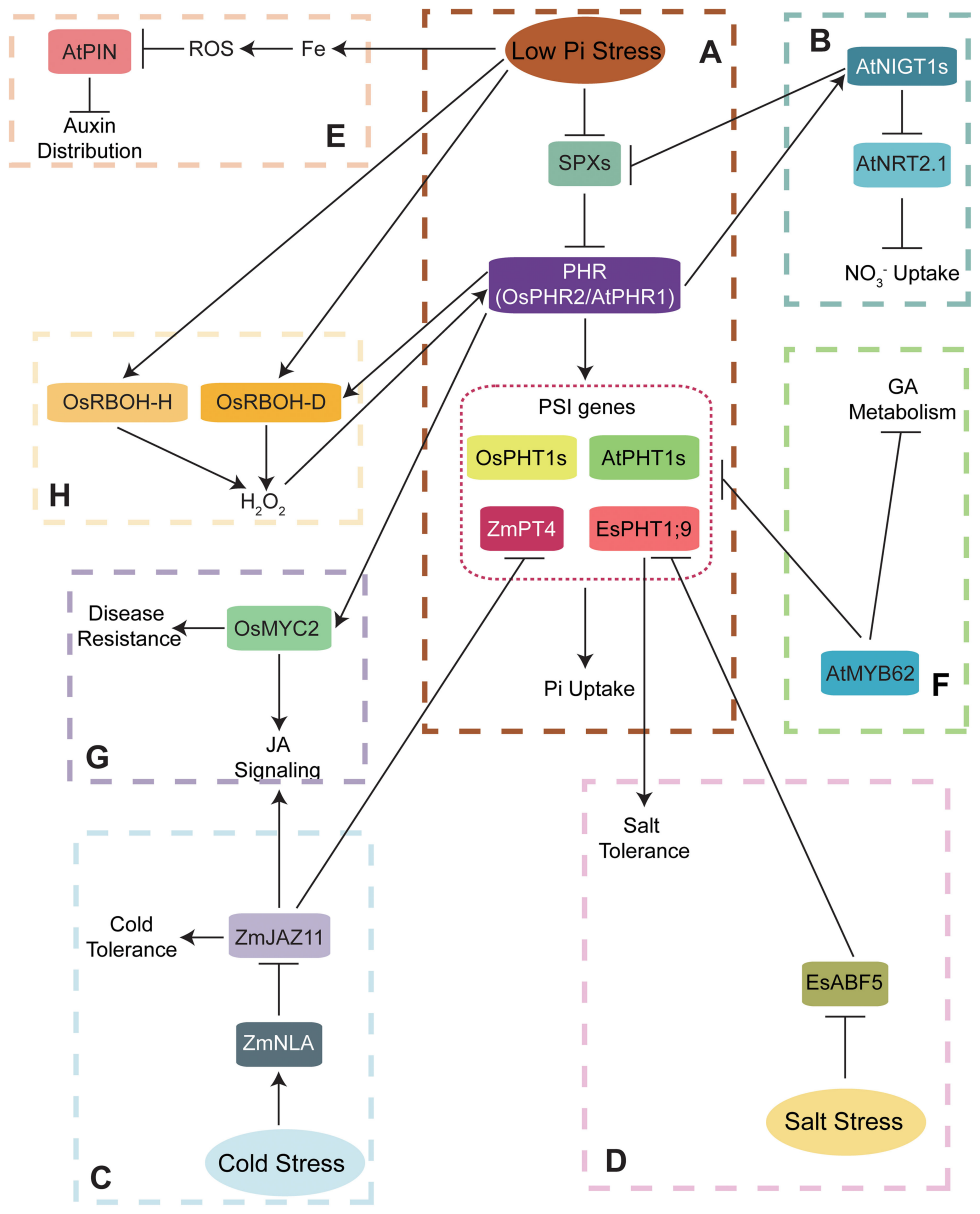
Crosstalk between low-Pi signaling and various signal pathways. **(A)** Pi signaling; **(B)** Nitrate signaling; **(C)** Cold stress signaling; **(D)** Salt stress signaling; **(E)** Auxin signaling; **(F)** Gibberellin signaling; **(G)** Jasmonic acid and disease resistance signaling; **(H)** Hydrogen peroxide signaling. Under low-Pi conditions, PHR2 drives the expression of PSI genes in rice and *Arabidopsis*, indicating that this regulatory mechanism is conserved. Through coordinated regulatory processes, PHR2 connects phosphate signaling with nutrient, hormonal, redox, immune, and stress responses, defining low-Pi signaling as a broad regulator of plant adaptation. Arrows mean positive regulation, while short bar lines represent negative regulation.

The Pi starvation signaling pathway does not function independently but instead extensively engages in crosstalk with other signaling pathways, including nitrogen (N) signaling, plant hormones, other abiotic stress signals, and redox signaling molecules (**Figure 3**). With respect to N–Pi signaling crosstalk, the GARP transcription factor NIGT1 is essential for balancing nitrate and phosphate signaling: AtNIGT1 is nitrate-induced but accumulates less under low-Pi environment, whereas PHR1 positively mediates *NIGT1*s, subsequently inhibiting NRT2.1 expression, thus reducing nitrate uptake and enhancing phosphate acquisition (**Figure 3**) (Maeda et al., 2018). Besides, NIGT1s repress the expression of *SPX* genes, including *SPX1*, in *arabidopsis* (**Figure 3**) (Ueda et al., 2021). In maize and *Arabidopsis*, NIGT1.2 directly activates phosphate transporter genes *PHT1;1* and *PHT1;4* while simultaneously suppressing NRT1.1, a nitrate transporter gene, under low-Pi conditions (Wang et al., 2020). Pi-deficient environments also promote ammonium absorption and rhizosphere acidification, triggering a STOP1-based regulatory network that enhances plant acquisition of sparingly soluble phosphate sources (Tian et al., 2021b).

Regarding the hormone signaling crosstalk, modulating exogenous auxin levels under low-Pi conditions affects phosphate uptake through altering root architecture. *MYB62* overexpression affects phosphate acquisition and root morphology while negatively regulating GA metabolism and signaling, highlighting a potential mechanistic link between low-Pi responses and GA signaling (**Figure 3**) (Devaiah et al., 2009). OsPHR2 confers disease resistance under Pi deficiency by activating *OsMYC2*, a pivotal component of the jasmonic acid signaling pathway, revealing the crosstalk between phosphate and jasmonic acid signaling (**Figure 3**) (Kong et al., 2021). A current study suggests that the impairment of primary root growth in *Arabidopsis* under low-Pi stress is primarily owing to Fe accumulation–induced ROS bursts, which repress PIN-mediated auxin transport, disturb auxin distribution, and block cell division (**Figure 3**) (Zhang et al., 2026).

Current research has revealed that the low-Pi stress signaling pathway also interacts with various abiotic stress signaling pathways, including salt and cold stress. *EsPHT1;9*, along with its promoter, confer increased tolerance to simultaneous salt and low-Pi stress in *Arabidopsis*, while *EsABF5* negatively modulates this tolerance through specific binding to the *EsPHT1;9* promoter, thereby demonstrating the interaction between phosphate and salinity stress signaling (**Figure 3**) (Lv et al., 2021). Additionally, NLA controls the trade-off between cold tolerance and phosphate uptake via differentially regulating *JAZ11* and *PT4*, and targeted engineering of this pathway has been shown to improve maize resilience to both cold and low-Pi stress (**Figure 3**) (Liao et al., 2026).

Recent studies indicate that the low-Pi signaling pathway interacts with redox signaling molecules, including H_2_O_2_ and H_2_S (**Figure 3**). *OsRBOH-D/H* is upregulated under Pi starvation to produce H_2_O_2_ in rice, oxidizing OsPHR2 and enhancing PSI gene expression as well as *OsRBOH-D* expression, thereby further amplifying H_2_O_2_ production and establishing a positive feedback loop that increases phosphate uptake and utilization, ultimately enhancing adaptation to low-Pi environments (**Figure 3**) (Meng et al., 2025). *DES1*-generated H_2_S stimulates *SQD1* expression in *Arabidopsis*, modulating root growth under low-Pi environment and partially mitigates primary root growth inhibition resulting from low-Pi stress (Liu et al., 2024). Pi signaling is tightly linked with nitrogen, hormone, stress and redox pathways through NIGT1, PHR1 and ROS-related modules. Ongoing studies are needed to explore how these interactions vary across tissues and species, particularly the way NIGT1–PHR1 and redox signaling act together in Pi uptake under combined stresses.

## 4 Conclusions and future perspectives

**Figure 4:**
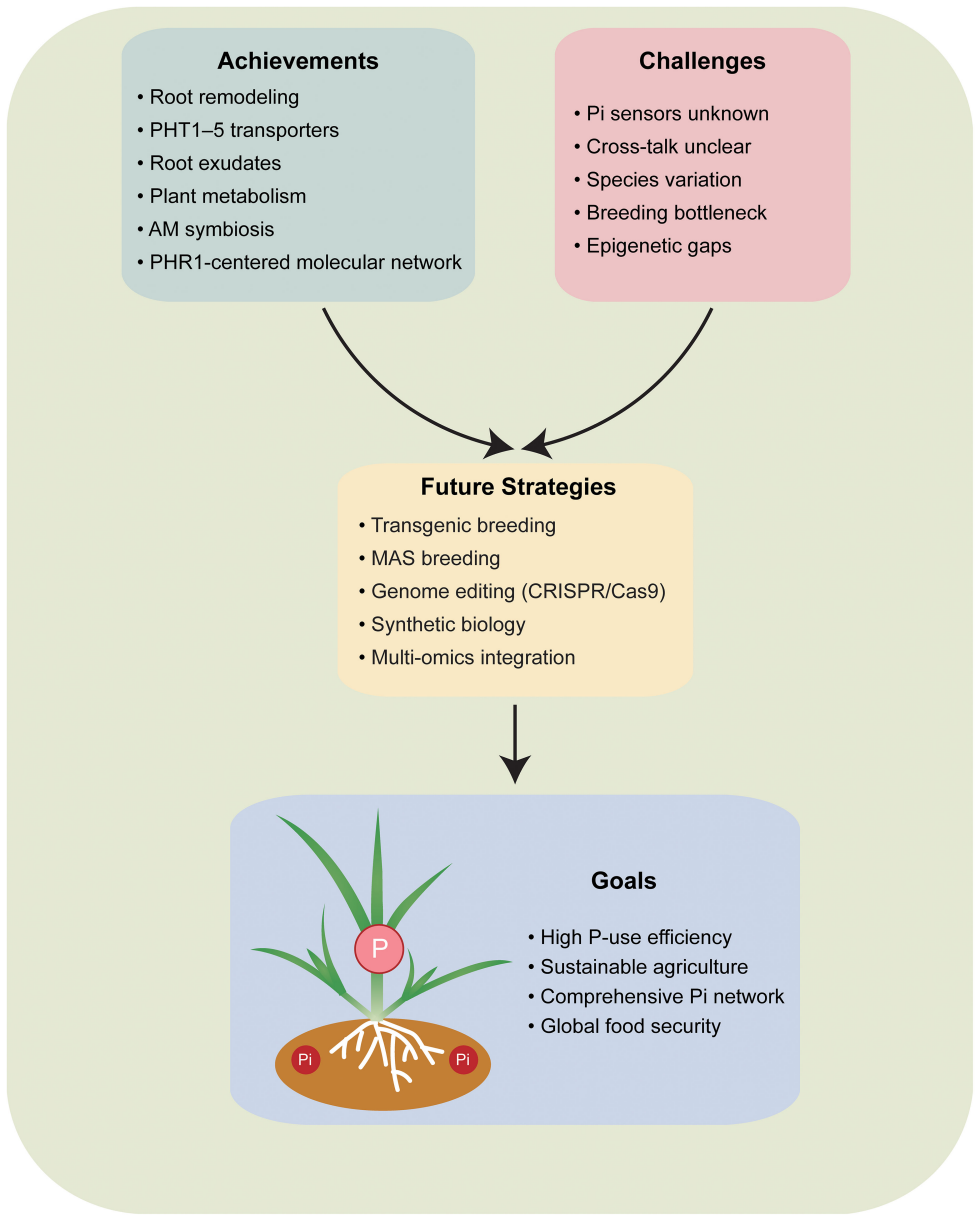
Current achievements, remaining challenges, proposed solutions and future goals for future low Pi adaptation investigation. Low-Pi adaptation includes root remodeling, Pi mobilization, metabolic reprogramming, and a PHR1-centered regulatory network. Remaining challenges include Pi sensing and signaling, cross-talk complexity, and genetic diversity among species. Future strategies such as precision breeding and multi-omics integration aim to improve P-use efficiency and support sustainable agriculture.

Significant advances have been made in illustrating plant adaptive responses to low-Pi stress, providing an initial understanding of the multi-layered response network spanning from physiological traits to molecular regulation (**Figure 4**). At the physiological level, adaptive strategies such as root system remodeling, improved root exudates secretion, and metabolic adjustments play essential roles in low-Pi stress adaptation in plants (**Figures 1** and **4**; **Supplementary Table 1**). At the level of molecular regulation, a PHR1-centered regulatory network integrates signals from a variety of transcription factor such as MYB, WRKY, and bHLH (**Figures 2** and **4**; **Supplementary Table 2**); SPX-domain proteins function as central sensors and controllers of phosphate signaling and homeostasis; and post-transcriptional modules, including miR399–*PHO2* and miR827–*NLA*/*SPX-MFS*, finely regulate phosphate uptake, transport, and distribution (**Figures 2** and **4**; **Supplementary Table 3**).

Although numerous key genes involved in Pi uptake, transport, and utilization have been identified, only a limited number of these genes have been successfully applied in commercial breeding programs. Future efforts should therefore place greater emphasis on bridging the gap between gene function discovery and crop improvement. Genetic engineering and synthetic biology approaches provide promising opportunities for improving crop phosphorus use efficiency (PUE). However, many currently identified candidate genes have been evaluated using constitutive promoters, such as the CaMV 35S or ubiquitin promoters. Although these strategies can enhance Pi acquisition or utilization, constitutive expression may also interfere with normal plant growth and development, resulting in reduced biomass accumulation or yield penalties, thereby limiting their practical application (Wang et al., 2026). Therefore, the application of tissue-specific promoters, Pi-responsive promoters, or inducible regulatory systems to achieve precise spatial and temporal control of gene expression represents a potential strategy to overcome these limitations.

Notably, a large proportion of currently characterized PUE-related genes have been identified from model species, whereas their functional validation and utilization in major crops, such as wheat, maize, and soybean, remain relatively limited. Future research should exploit comparative genomics and ortholog identification approaches to transfer conserved Pi regulatory mechanisms from model species to non-model crops. The SPX–PHR signaling module represents a highly conserved core regulatory hub of Pi homeostasis, offering an important foundation for cross-species functional validation and crop improvement. For instance, homologous cloning of PUE-associated genes from other crops, followed by functional characterization, may accelerate the utilization of conserved beneficial genes to improve Pi efficiency in diverse crop species. In rice, overexpression of *OsPSTOL1* has been shown to significantly improve grain yield under P-deficient environment (Gamuyao et al., 2012). Homologous cloning approaches can be employed to identify and utilize corresponding genes in other crops. Moreover, heterologous expression of key regulatory genes from model plants may enhance Pi acquisition and utilization in crops. For example, introducing *AtPAP15* into soybean increased phosphatase and phytase activities, which contributed to more efficient acquisition of phosphorus from organic sources (Wang et al., 2009b). These findings suggest that combining conserved regulatory modules with species-specific genetic resources could advance the application of Pi-efficiency genes in crop improvement.

Beyond engineered strategies, naturally occurring genetic variation provides an important resource for understanding Pi adaptation mechanisms and developing Pi-efficient crops. Future studies can integrate genome-wide association studies (GWAS), linkage mapping, and high-throughput phenotyping platform to identify key quantitative trait loci (QTLs) controlling Pi uptake, transport, and utilization across diverse germplasm collections. Functional markers tightly linked to favorable alleles can subsequently be developed and applied in marker-assisted selection (MAS) breeding programs. More importantly, the construction of near-isogenic lines (NILs) can help minimize linkage drag, enabling more accurate evaluation of the contribution of elite alleles to Pi-use efficiency and promoting their utilization in breeding. In our previous study, a natural 50-bp deletion in the *HvSPX4* promoter reduced its expression and improved barley growth under Pi deficiency (Zhang et al., 2025). This natural variation represents a potential molecular marker for MAS-based breeding of Pi-efficient barley varieties. Additionally, for complex traits governed by multiple minor-effect loci, pyramiding favorable alleles may further improve crop PUE.

Genome editing technologies provide additional opportunities for precise improvement of crop phosphorus efficiency. Compared with conventional transgenic approaches, CRISPR/Cas-based systems enable targeted modification of key regulatory genes, promoter regions, and regulatory networks, allowing more refined optimization of endogenous gene functions. In rice, targeted deletion of a negative regulatory W-box element in the *OsPHO1;2* promoter enhanced *OsPHO1;2* expression, promoted Pi translocation from roots to shoots, and improved yield-related traits without compromising grain quality (Maurya et al., 2025). In addition, precise modulation of gene activity, rather than complete gene disruption or constitutive activation, can help maintain the balance between Pi acquisition and plant growth. A tissue-specific CRISPR/Cas9 knockout of *OsPHO2* improved low-P tolerance and grain yield by optimizing Pi homeostasis while avoiding the growth defects observed in whole-plant mutants (Wei et al., 2026). Furthermore, editing Pi-responsive regulatory regions may provide a strategy for fine-tuning gene expression patterns by activating beneficial genes under Pi-deficient conditions, thereby minimizing the negative effects associated with constitutive expression. In the future, integrating genome editing with synthetic biology approaches may enable the construction of artificial Pi-responsive regulatory circuits, facilitating coordinated regulation of multiple genes involved in Pi uptake, transport, and metabolism. Such strategies may accelerate the development of next-generation crops with enhanced phosphorus-use efficiency and reduced reliance on phosphate fertilizers.

## Supplementary material

Supplementary Figure 1 Distribution of available P in global arable land in relation to agronomic thresholds (McDowell et al., 2024). The agronomic threshold for optimum production in arable land is 15 mg kg^−1^.

Supplementary Table 1 Phosphate transporter (PHT) families in rice and *arabidopsis.*

Supplementary Table 2 SPX domain-containing proteins in rice and *arabidopsis.*

Supplementary Table 3 TFs responding to Pi starvation.
