# Supplementary material for: Adaptation mechanisms of low-phosphorus stress in plants: physiological responses, molecular regulation, and future perspectives
Source: Front Plant Sci. 2026 Jul 30;17:1877859. doi: 10.3389/fpls.2026.1877859 (PMC13470894; doi:10.3389/fpls.2026.1877859)
Supplement: Supplementary file 1 [file Image1.pdf]

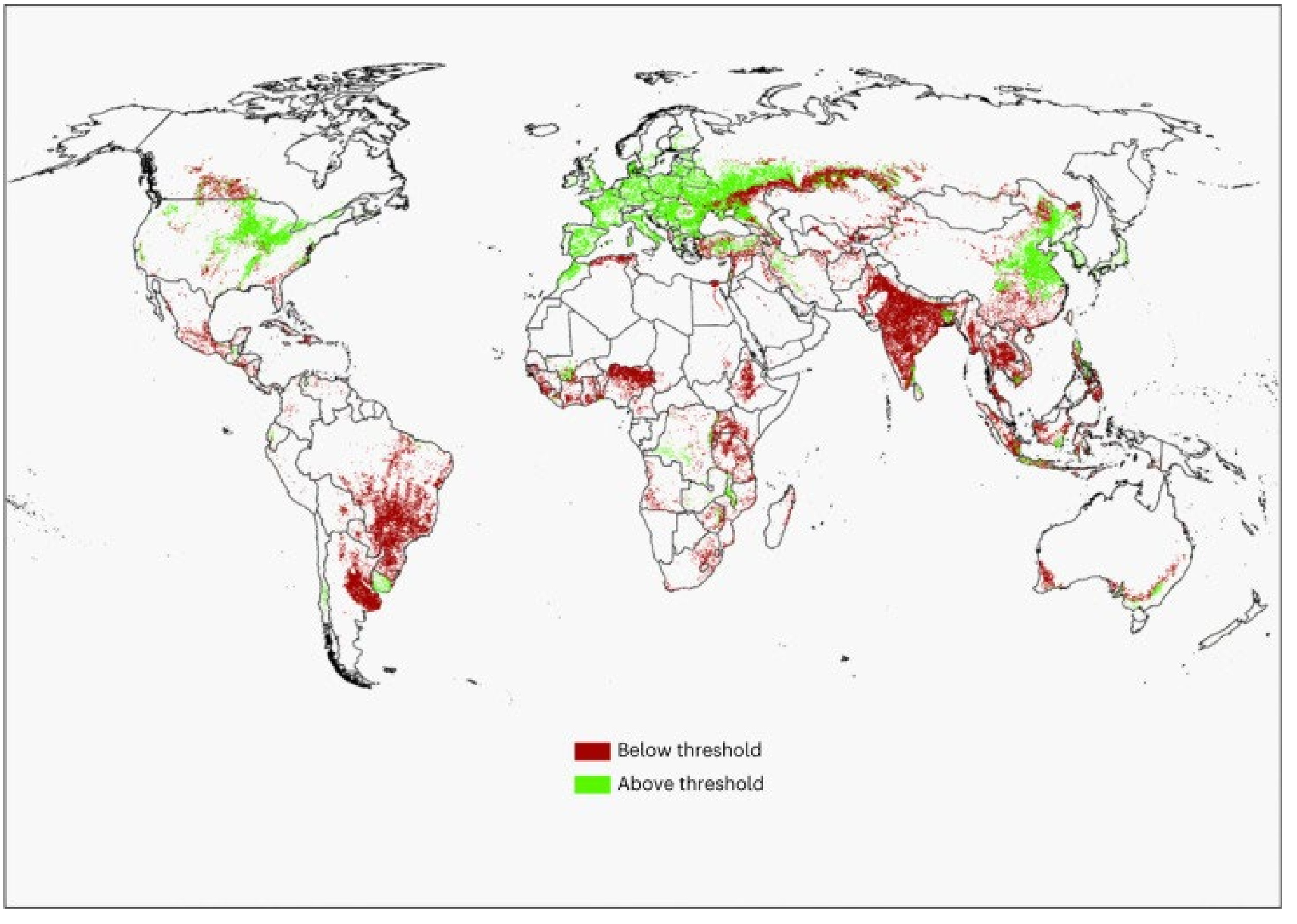

Supplementary Figure 1. Distribution of available P in global arable land in relation to agronomic thresholds (McDowell et al., 2024)

The agronomic threshold for optimum production in arable land is 15 mg kg^{-1}.
